# Performance of Different Herbicides in Dry-Seeded Rice in Bangladesh

**DOI:** 10.1155/2014/729418

**Published:** 2014-02-06

**Authors:** Sharif Ahmed, Bhagirath Singh Chauhan

**Affiliations:** ^1^Bangladesh Agricultural University, 2202 Mymensingh, Bangladesh; ^2^International Rice Research Institute, Los Baños, DAPO Box 7777, 4031 Metro Manila, Philippines

## Abstract

A field study was conducted in the boro season of 2011-12 and aman season of 2012 at Jessore, Bangladesh, to evaluate the performance of sequential applications of preemergence herbicides (oxadiargyl 80 g ai ha^−1^, pendimethalin 850 g ai ha^−1^, acetachlor + bensulfuranmethyl 240 g ai ha^−1^, and pyrazosulfuron 15 g ai ha^−1^) followed by a postemergence herbicide (ethoxysulfuron 18 g ai ha^−1^) in dry-seeded rice. All evaluated herbicides reduced weed density and biomass by a significant amount. Among herbicides, pendimethalin, oxadiargyl, and acetachlor + bensulfuranmethyl performed very well against grasses; pyrazosulfuron, on the other hand, was not effective. The best herbicide for broadleaf weed control was oxadiargyl (65–85% control); pendimethalin and acetachlor + bensulfuraonmethyl were not effective for this purpose. The best combination for weed control was oxadiargyl followed by ethoxysulfuron in the boro season and oxadiargyl followed by a one-time hand weeding in the aman season. Compared with the partial weedy plots (hand weeded once), oxadiargyl followed by ethoxysulfuron (4.13 t ha^−1^) provided a 62% higher yield in the boro season while oxadiargyl followed by a one-time hand weeding (4.08 t ha^−1^) provided a 37% higher yield in the aman season.

## 1. Introduction

In many Asian countries, growers recently started to shift their rice cultivation practices from the traditional puddled-transplanted rice (PTR) to dry-seeded rice (DSR). Puddling or repeated tillage under wet conditions is a labor-, water-, and energy-intensive method [1, 2]. The cost of farm labor is increasing because workers engaged in agriculture are now moving to the cities for employment in nonfarm jobs, such as in the textile and garment industries. As a result, it is difficult to find labor at the peak period of transplanting, which delays transplanting and often leads to the use of aged seedlings. At the peak of the transplanting period, the labor demand pushes wage above normal rates.

Conventional rice production systems, such as PTR, require large quantities of water. It has been estimated that seasonal water input for typical PTR ranges from 660 to 5280 mm, depending on the growing season, climatic conditions, soil type, and hydrological condition [3]. Of the total amount of water required for rice culture in a season, about 30% is used for land preparation in puddled systems [4]. Water for agriculture is becoming increasingly scarce worldwide, including Bangladesh.

The key concerns, therefore, are how the water requirement of rice culture can be reduced and how farmers can avoid puddling and transplanting operations without yield loss. The development of alternative methods that are more water-efficient and less labor-intensive is thus important to enable farmers to produce more with less cost. These factors demand a major shift from PTR production to dry seeding of rice in irrigated areas. DSR has the potential to reduce water and labor use compared with the conventional transplanted rice by eliminating the puddling phase and not requiring continuous standing water.

DSR systems have several advantages over PTR systems. However, weeds are a serious problem in DSR and reduce the productivity of the system [5–7]. Weeds are more problematic in DSR than in PTR because, without standing water to suppress weed emergence and growth, crop and weed emerge simultaneously [5, 6, 8]. Yield losses in DSR systems can go as high as 90% if control measures are not taken [9]. Timely weed control is therefore crucial in improving the productivity and profitability of DSR [9].

Various weed management strategies, such as thorough land preparation; high seeding rate; appropriate fertilizer application; optimum water management; and manual, mechanical, and chemical weed control are used in DSR cultivation [10, 11]. Manual weeding is common in Asian countries, but its use is decreasing because of labor scarcity at the critical time of weeding and increasing labor costs [12]. In addition, some weeds (e.g., Echinochloa colona (L.) Link. and E. crus-galli (L.) Beauv.) at their early growth stages look similar to rice seedlings, making hand weeding (HW) difficult in DSR. Chemical methods of weed control are therefore the most practical and cost-efficient [8, 13].Several preemergence (e.g., pendimethalin, oxadiazon, oxdiargyl, and pyrazosulfuron) and postemergence herbicides (e.g., bispyribac-sodium, azimsulfuron, penoxsulam, fenoxaprop, ethoxysulfuron, and 2,4-D) are now available in various Asian countries and have been reported to provide effective weed control [11].

In a previous study, the sequential preemergence application of pendimethalin (1000 g ai ha^−1^) followed by postemergence application of bispyribac-sodium (30 g ai ha^−1^) at 15 days after sowing (DAS) was found best for the control of weeds in DSR [14]. In another study, the best result was found with the application of oxadiazon (750 g ai ha^−1^) applied at 2 DAS followed by fenoxaprop + ethoxysulfuron (45 g ai ha^−1^) applied at 28 DAS [12]. Results from different studies reveal that no single herbicide can control all weeds effectively in DSR systems. Moreover, the continuous use of a single herbicide over a long period of time may develop herbicide resistance in weeds and shifts in weed flora [2, 15]. The right selection of herbicides is thus very important in DSR.

In Bangladesh, chemical weed control is becoming popular because of labor scarcity and also because it costs less than HW [16, 17]. However, information on the performance of herbicides is only available for transplanted rice and not for DSR systems. This study was therefore conducted to evaluate the performance of sequential applications of various preemergence herbicides followed by postemergence herbicides in DSR—the first was study conducted in Bangladesh for this purpose.

## 2. Materials and Methods

**Figure 1:**
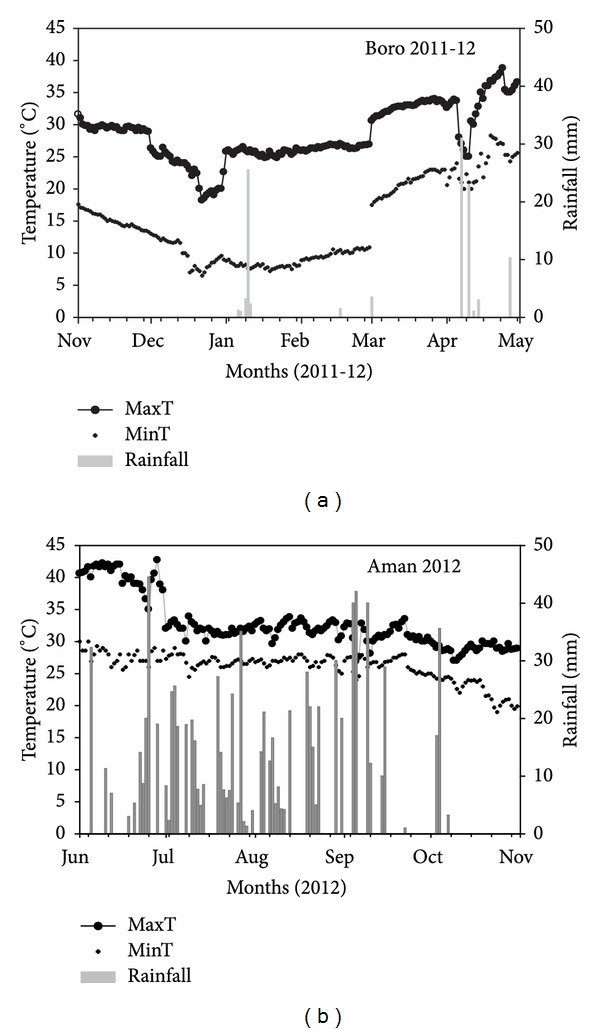
Maximum and minimum temperatures and total rainfall (mm) recorded at the experimental site in the boro and aman seasons of 2011-12.

Field experiments were conducted at the research farm of the Regional Agricultural Research Station of the Bangladesh Agricultural Research Institute (23°11′ N, 89°14′ E, and 6.71 m above mean sea level) in Jessore, Bangladesh, in the boro (dry) season of 2011-12 and in the aman (wet) season of 2012. The area belongs to agroecological zone number 11, known as the High Ganges River Floodplain. The climate in the area is subtropical, with an average annual rainfall of 1590 mm (90% of which falls from May to September), minimum temperatures of 6–9°C in January, and maximum temperatures of 36–44°C in April-May. Amount of rainfall and the minimum and maximum temperatures recorded at the experimental site during the experimental periods are presented in Figure 1. The weather data were collected from the Regional Agricultural Research Station located 300 m from the experimental field. The soil in the experimental field at 0–15 cm depth was clay loam in texture (37.8% clay, 31.5% sand, and 30.7% silt) with a bulk density of 1.58 g cm^−3^, pH of 7.8, and organic carbon content of 1%.

Prior to the start of the experiment, the field was leveled using a laser leveler. Different rice varieties are recommended for use in different seasons, and from which BRRI dhan28 (140 d duration) and BRRI dhan49 (135 d duration) were selected for the boro and aman seasons, respectively. In both seasons, certified seeds were used (source: Bangladesh Agriculture Development Corporation, Jessore, Bangladesh). The experiments in each season were arranged in a randomized complete block design with three replications. Unit plot size was 18 (6 × 3) m^2^.

Ten weed control treatments were used in each season: (i) weed-free, (ii) partial weedy, (iii) oxadiargyl (80 g ai ha^−1^ applied at 2 DAS) followed by ethoxysulfuron (18 g ai ha^−1^ applied at 21 DAS), (iv) pendimethalin (850 g ai ha^−1^ applied at 2 DAS) followed by ethoxysulfuron (18 g ai ha^−1^ applied at 21 DAS), (v) acetachlor + bensulfuranmethyl (240 g ai ha^−1^ applied at 2 DAS) followed by ethoxysulfuron (18 g ai ha^−1^ applied at 21 DAS), (vi) pyrazosulfuron (15 g ai ha^−1^ applied at 2 DAS) followed by ethoxysulfuron (18 g ai ha^−1^ applied at 21 DAS), (vii) oxadiargyl (80 g ai ha^−1^ applied at 2 DAS) followed by a one-time hand weeding (HW) at 35 DAS, (viii) pendimethalin (850 g ai ha^−1^ applied at 2 DAS) followed by a one-time HW at 35 DAS, (ix) acetachlor + bensulfuranmethyl (240 g ai ha^−1^ applied at 2 DAS) followed by a one-time HW at 35 DAS, and (*x*) pyrazosulfuron (15 g ai ha^−1^ applied at 2 DAS) followed by a one-time HW at 35 DAS.

Herbicides were applied using a knapsack-sprayer fitted with three flat fan nozzles on a boom, delivering 350 L of solution ha^−1^. In weed-free plots, weeds were removed manually (5 times in the aman season and 7 times in the boro season). In the partial weedy plots, HW was done once at 35 DAS; weeds were allowed to grow before and after it. Weeds allowed to grow throughout the season may result in an almost 100% yield loss in DSR systems [9]. In addition, farmers in irrigated areas rarely leave their rice fields infested with weeds throughout the season.

Dry rice seeds were sown at a rate of 40 kg ha^−1^ and with row spacing of 20 cm, using a power tiller operated seed drill fitted with a fluted-type seed-metering device. The crop was planted on November 22, 2011 and June 17, 2012 in the boro and aman seasons, respectively. Fertilizers were applied at the rate of 160-20-60-12-2.2 kg ha^−1^ of  N, P, K, S, and Zn, respectively, in the boro season; and 120-15-48-12-2.2 kg ha^−1^ of  N, P_,_ K, S, and Zn, respectively, in the aman season. All P, K, S, and Zn were applied immediately before sowing. Fertilizer N was applied in four equal splits (25% each) at 14 DAS, at the start of tillering, at maximum tillering, and at booting stage.

Light irrigation was supplied just after sowing and the field was kept saturated up to 40 DAS in the boro season and up to 20 DAS in the aman season, after which irrigation was supplied based on tensiometer readings using a threshold value of 15 kPa at 15 cm soil depth. Water was maintained at 5 cm depth with each irrigation. In the boro season, the rice plants were severely infested with blast and leaf spots. Fungicide tebuconazole + trifloxystrobin 75 WP (300 g ai ha^−1^) was thus applied at 35 and 70 DAS. Fipronil 3 G (10 kg ai ha^−1^) was applied at 70 DAS in the boro season and 40 DAS in the aman season to control stem borers.

Rice plant density was counted at 14 DAS from four randomly selected 1 m row lengths in each plot. To evaluate the efficacy of preemergence herbicide, weed density was counted groupwise (i.e., grasses, broadleaves, and sedges) at 20 DAS (i.e., before the application of postemergence herbicide). These data were collected only in the aman season and weed biomass data was not measured because of the very small size of weed seedlings.

To evaluate the efficacy of postemergence herbicide, weed density and weed biomass were measured at 35 DAS and at anthesis. At each sampling time, two 40 cm × 40 cm quadrats were placed randomly in each plot. Weeds were then collected from each quadrat, segregated by species, and counted. Biomass was measured species-wise after samples were oven-dried at 70°C for 72 h. Rice tillers were collected from the same quadrats used for weed sampling and then counted, and rice biomass was measured. At harvest, the number of rice panicles was counted from four randomly selected 1 m row lengths in each plot. The number of grains per panicle (filled and unfilled spikelets) was counted by a random sampling of 20 panicles per plot. Rice grain yield was determined from the harvested area of 8.8 (4.0 × 2.2 m) m^2^. Grain yield was converted to t ha^−1^ at 14% moisture content, and 1000-grain weight was measured.

Data were analyzed using analysis of variance (ANOVA) to evaluate differences among treatments. Means were separated using least significant differences (LSD) at 5% level of significance using CropStat 7.2 (IRRI, Philippines). Weed density and biomass data were subjected to square-root transformation ($\sqrt{x+0.5}$).

## 3. Results and Discussion

### 3.1. Effect of Herbicides on Rice Plant Density

**Figure 2:**
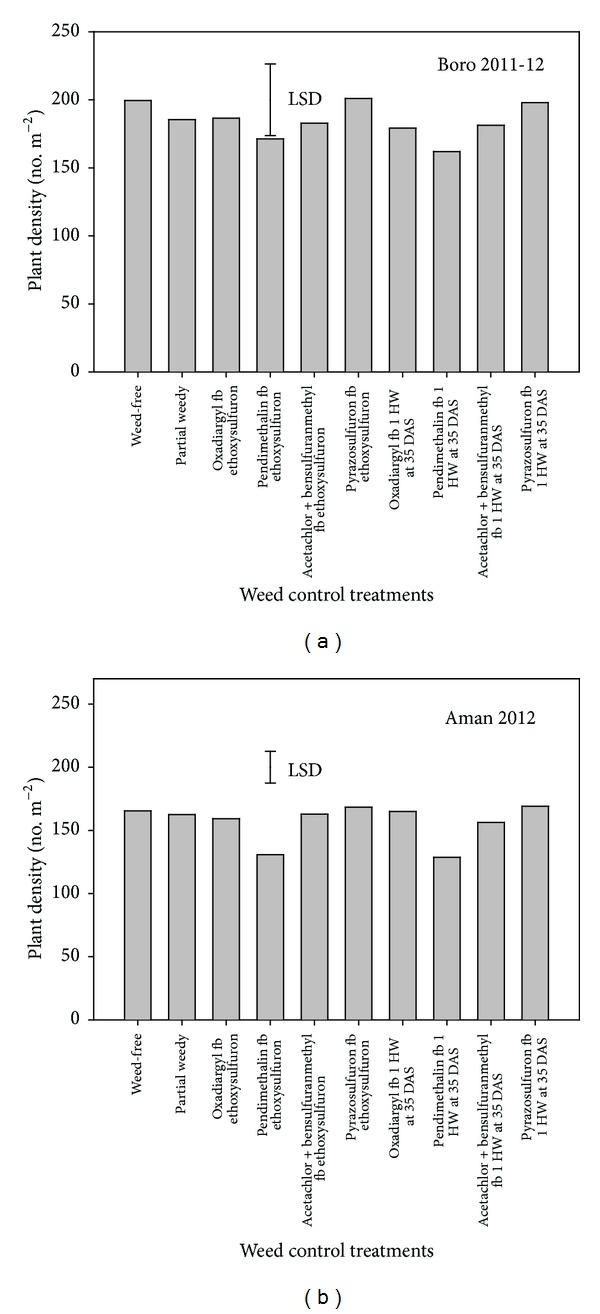
Effect of weed control treatment on rice plant density (number m^−2^) at 14 days after sowing.

Weed control treatments affected rice plant density in the aman season but not in the boro season. Plant density range was 162–201 plants m^−2^ in the boro season and 128–169 plants m^−2^ in the aman season (Figure 2). In both seasons, the lowest plant density was found in plots treated with pendimethalin. Pendimethalin-treated plots had 14% and 22% lower plant densities in the boro and aman seasons, respectively, compared with the plots that were not treated with any herbicide. Other herbicide-treated plots had similar plant densities in both seasons. In the aman season, heavy rains occurred after herbicide application (Figure 1), which could be the main cause of low plant density in the pendimethalin-treated plots. No heavy rains occurred after herbicide application in the boro season. The results suggest that pendimethalin can cause phytotoxicity if heavy rainfall occurs after application of herbicide. Similar results were reported for oxadiazon herbicide, in which rice plant density was lower compared with nontreated plots when heavy rains occurred immediately after herbicide application [18]. These studies suggest that soil water content is an important factor that can influence herbicide phytotoxicity in rice. Such toxicity can result in poor crop establishment, especially where low seeding rates are used.

### 3.2. Effect of Weed Control Treatments on Weed Density and Biomass

Weed species varied with the growing seasons. Some species were not common in both seasons. For example,* Amaranthus spinosus* L., *Anagalis arvensis *L.,* Cleome rutidosperma *DC., and *Cynodon dactylon *(L.) Pers. were present only in the boro season and* Ageratum conyzoides *L. was present only in the aman season. Such variations might be the result of the seasonal adaptation of weeds. In addition, nonrice crops were grown before the start of the experiment. This may be another reason for the variation. In the boro season, temperatures were low up to 60 DAS but it later increased (Figure 1). Because of low initial temperatures, crop and weed growth was slow. However, the growth of some species (e.g., *Cyperus rotundus *L., *Cynodon dactylon*, and *Cleome rutidosperma*) was unaffected by the low temperatures and severely suppressed crop growth at its early stages in the boro season. Another interesting observation was that some weed species (e.g., *A. arvensis*,* Galinsoga ciliate *Blake, and *Phyllanthus niruri *L.) emerged later than usual, which could be their strategy to “escape” the application of preemergence herbicides.

The dominant weed species in the experiments were *Celosia argentea* L., *Cyperus rotundus*, *Dactyloctenium aegyptium* (L.) Willd., *Digitaria ciliaris* (Retz.) Koel., *E. colona*, *Eleusine indica* (L.) Gaertn, *G. ciliate*, and *P. niruri*. The application of preemergence herbicides significantly reduced weed density, compared with the control plots (partial weedy). At 20 DAS (before application of postemergence herbicide), plots treated with preemergence herbicides had 45–70% lower weed density than the partial weedy plots (Table 1).

Among the herbicide treatments, oxadiargyl-treated plots had the lowest total weed density (157 plants m^−2^), followed by those treated with acetachlor + bensulfuranmethyl (181 plants m^−2^). The highest weed density was recorded in the plots treated with pyrazosulfuron. In terms of weed groups, the lowest grass density was recorded in the plots treated with pendimethalin (88% less than in the partial weedy check) and the highest was recorded in the plots treated with pyrazosulfuron (only 10% less than in the partial weedy check). Against broadleaved and sedges, acetachlor + bensulfuraonmethyl, oxadiargyl, and pyrazosulfuron performed well (63–70% fewer broadleaved weeds and 54–70% fewer sedges compared to the partial weedy check) while pendimethalin performed poorly (suppressed only 37% and 18% of broadleaved and sedges, resp.). The observed effectiveness of pendimethalin in the control of grasses and its poor control of sedges and broadleaved weeds are consistent with the findings of previous published studies [8, 16, 17, 19].

At 35 DAS (after application of postemergence herbicides and before HW), total weed density and weed biomass were greatly affected by herbicide treatments. Plots treated with preemergence herbicides had 39–76% and 23–45% lower weed densities (326 and 293 plants m^−2^ in the boro and aman seasons, resp.) compared with the partial weedy check (Tables 2 and 3). However, the values were 66–88% and 46–65%, respectively, when plots were treated with both pre- and postemergence herbicides.

Among the herbicide treatments, oxadiargyl-treated plots performed best, followed by those treated with pendimethalin and acetachlor + bensulfuranmethyl. Poor performance was observed in the pyrazosulfuron-treated plots. A similar trend was found for total weed biomass (Tables 4 and 5). Plots that were treated with only preemergence herbicides had 50–66% and 29–57% less weed biomass than the partial weedy plots in the boro and aman seasons, respectively. Plots that received both pre- and postemergence herbicides had 70–80% and 44–67% less weed biomass in the boro and aman seasons, respectively. The effect of herbicides on individual weed density and biomass was significant in both seasons except on *C. dactylon*, suggesting that applying herbicide was not effective against this weed species. Oxadiargyl was superior to other herbicides in reducing weed density and biomass of several individual weed species, including *E. colona, Ageratum conyzoides*,* Amaranthus spinosus, Cleome rutidosperma, *and *Celosia argentea*. The next best treatment was pendimethalin, which suppressed all weeds that were suppressed by oxadiargyl, except *C. argentea *and *P. niruri*. However, both herbicides were ineffective against* C. rotundus *in both seasons.

Plots treated with both pre- and postemergence herbicides always had less total weed density and biomass than those that were treated with only preemergence herbicides. For example, the plots that received only preemergence herbicides had a weed density range of 70–182 and 157–233 plants m^−2^ in the boro and aman seasons, respectively. In the plots that received both pre- and postemergence herbicides, weed density range was 43–107 and 84–152 plants m^−2^, respectively. The same trend was observed in weed biomass, which indicates that the postemergence herbicide ethoxysulfuron significantly lowered weed density and biomass. Ethoxysulfuron suppressed a range of broadleaved weed species (e.g., *Ageratum conyzoides, Amaranthus spinosus, Cleome rutidosperma, Celosia argentea*, and *P. niruri*) and sedges (e.g., *C. rotundus*) but not grasses (e.g., *E. colona, D. ciliaris*,and *C. dactylon*). Ethoxysulfuron has been reported to be effective in controlling a wide range of broadleaved weeds as well as sedges [20, 21]. Our results are also supported by a previous study on the furrow-irrigated raised-bed DSR, in which ethoxysulfuron (18 g ai ha^−1^) applied at 21 DAS was effective in controlling broadleaved weeds [8].

Weed density and biomass, as affected by different weed control treatments at anthesis, are given in Tables 6–9. From the data, it is clear that all herbicide treatments resulted in lower weed density and biomass compared to the partial weedy plots (i.e., HW at 35 DAS). Similar with observations made at 35 DAS, total weed density and biomass were also greatly affected by weed control treatments at anthesis. Considering the total weed density, plots treated with pre- followed by postemergence herbicides always had lower weed density than those treated with preemergence herbicides followed by a one-time HW at 35 DAS (Tables 6 and 7). Weed biomass maintained a similar trend as weed density in the boro season. In the aman season, however, plots that received preemergence herbicides followed by a one-time HW had lower weed biomass than those that received pre- and postemergence herbicides (Tables 8 and 9).

In the aman season, crop vegetative growth went very fast at the early growth stages. Rice in the plots that received a one-time HW at 35 DAS suppressed weed growth at later stages. In the boro season, however, rice growth was very poor up to 65 DAS because of low temperatures. Sequential application of pre- and postemergence herbicides reduced weed biomass by 31–55% and 20–52% in the boro and aman seasons, respectively; and preemergence herbicide application followed by a one-time HW reduced biomass by 15–40% and 40–62% in the respective seasons, all compared with the partial weedy check (i.e., one-time HW).

Among the herbicide treatments, plots treated with oxadiargyl followed by ethoxysulfuron reduced total weed density by 65–70% compared with the partial weedy plots (378–477 plants m^−2^) (Tables 6 and 7). The next best treatments were pendimethalin followed by ethoxysulfuron (60–62% reduction in weed density) and acetachlor + bensulfuranmethyl followed by ethoxysulfuron (52–55% reduction in weed density). Compared with the partial weedy plots, those treated with pyrazosulfuron followed by ethoxysulfuron suppressed weed density by only 39–48%.

### 3.3. Effect of Weed Control Treatments on Yield and Yield-Contributing Parameters

Yield and yield-contributing parameters, except 1000-grain weight, were highly influenced by weed control treatments and the results were consistent in both seasons (Tables 10 and 11). The highest panicle numbers (253–347 m^−2^) were recorded in the weed-free plots. Panicle number was always lower in the herbicide-treated plots than in the weed-free plots and always higher than in the partial weedy plots. In the boro season, the highest panicle numbers (281–316 m^−2^) were recorded in the plots that received pre- followed by postemergence herbicides. In the aman season, these values (204–236 panicles m^−2^) were highest when the plots received preemergence herbicides followed by a one-time HW. Among herbicide treatments, the highest panicle numbers (230–315 m^−2^) were recorded in the oxadiargyl-treated plots. The next best herbicide treatment was pendimethalin, with panicle numbers (230–301 m^−2^) comparable to that of the oxadiargyl-treated plots. The lowest panicle numbers were recorded in the pyrazosulfuron-treated plots in both seasons.

The number of spikelets panicle^−1^ was consistent across the herbicide-treated plots in the boro season but it significantly differed in the aman season (Tables 10 and 11). Herbicide-treated plots always had a higher number of spikelets panicle^−1^ (5–26% higher) than the partial weedy plots and always had a lower number than the weed-free plots (3–21% lower).

Grain yield was strongly influenced by weed control treatments (Tables 10 and 11). The highest yield (4.76–4.98 t ha^−1^) was obtained in the weed-free plots. Herbicide-treated plots always yielded more than the partial weedy plots. Among the herbicide treatments, the highest yield was recorded in the plots treated with oxadiargyl followed by ethoxysulfuron (4.13 t ha^−1^) in the boro season and oxadiargyl followed a one-time HW (4.1 t ha^−1^) in the aman season. Pendimethalin-treated plots produced a similar grain yield (4.1 t ha^−1^). The plots treated with pyrazosulfuron produced the lowest yield in both seasons. Compared with the control treatment, herbicide-treated plots produced a 20–62% higher yield when treated with both pre- and postemergence herbicides; these values were 30–41% when plots received only preemergence herbicide and a one-time HW. Although herbicide treatments resulted in a yield advantage over the control treatment, none of the herbicide treatments reached the yield levels from the weed-free plots. Grain yield from the best-performing herbicide treatment was 13–18% lower than that from the weed-free plots, suggesting that there is a considerable scope to increase yield with improved weed control practices in DSR. In the boro season, yield was higher in the plots treated with preemergence followed by postemergence herbicides but the results were not consistent in both seasons. In the aman season, vegetative growth started early. In the plots that received preemergence herbicide followed by a one-time HW, canopy closure occurred fast, which suppressed weed growth at the later stages. These results suggest that, in the aman season, application of preemergence herbicide followed by HW was better than applying both preemergence and postemergence herbicides. However, these results also depend on others factors, such as efficacy of the postemergence herbicide used, weed flora in the field, rice variety used, and soil and climatic conditions. Information to support these results is not available in the literature.

Our study shows that even after the use of preemergence followed by postemergence herbicides at 21 DAS or preemergence followed by a one-time HW, complete weed control cannot be achieved. In addition, manual weeding is becoming less popular due to high labor wages; it will be difficult to find labor for weeding in the future. Therefore, additional research needs to be conducted on the performance of combinations of different pre- and postemergence herbicides, application timings, and herbicide doses across various seasons and environmental conditions.

**Figure 3:**
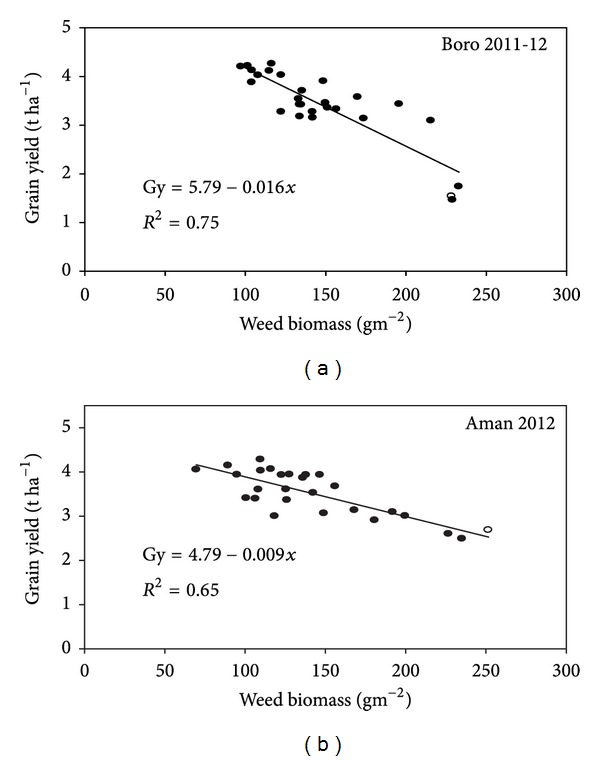
The relationship between grain yield and weed biomass.

We found that some weed species emerged later in the season (e.g., *A. arvensis*, *P. niruri*, and *G. ciliata*) and thus escaped the postemergence herbicide application or the HW at 35 DAS. Previous studies report that weeds emerging later in the season may not reduce rice yield significantly but add seeds to the soil seed bank and result in heavy weed infestation in subsequent seasons [22, 23]. In both seasons, we found a significant and negative linear correlation between grain yield and weed biomass at anthesis (Figure 3). The relationship was slightly stronger in the boro season (*R* ^2^ = 0.75) than in the aman season (*R* ^2^ = 0.65). These results are supported by previous studies in India [10] and the Philippines [18], both of which found that grain yield was negatively correlated with weed biomass.

In conclusion, it is clear that weeds are a serious problem in DSR production systems and herbicides are the essential tool to control them. However, continuous use of a single herbicide over a long period may cause the development of herbicide-resistant weed biotypes. Therefore, it is crucial to evaluate herbicides and determine which are less harmful to the crop and have the ability to control a wide range of weeds. In addition, when many effective herbicides are available, farmers will have the option to rotate the use of different herbicides and will help reduce the risk of resistance developing in weeds and shifts in weed flora toward problematic weeds.

## Figures and Tables

**Table 1:** Effect of weed control treatments on weed density (number m^−2^) at 20 DAS (before application of post-emergence herbicide). Weed density data were subjected to square-root $\left(\sqrt{\left[x+0.5\right]}\right)$ transformation before analysis; original values are shown in parentheses.

Weed control treatments	Grasses	Broadleaf	Sedges	Total
Partial weedy	13.68 (187.5)	15.41 (238.5)	10.10 (102.1)	22.98 (528.1)
Oxadiargyl	6.80 (45.8)	8.98 (80.2)	5.62 (31.3)	12.56 (157.3)
Pendimethalin	5.04 (25.0)	12.31 (151.0)	9.15 (83.9)	16.13 (259.9)
Acetachlor + bensulfuranmethyl	6.75 (45.3)	9.41 (88.5)	6.90 (47.4)	13.48 (181.2)
Pyrazosulfuron	13.00 (168.8)	8.46 (71.4)	5.97 (35.4)	16.62 (275.6)
LSD_0.05_	1.29	1.64	1.55	1.39
*P* value	<0.001	<0.001	0.001	<0.001

DAS: days after sowing; LSD: least significant difference at 5% level of significance; *P*: probability.

**Table 2:** Effect of weed control treatments on weed density (number m^−2^) at 35 DAS in the boro season. Weed density data were subjected to square-root $\left(\sqrt{\left[x+0.5\right]}\right)$ transformation before analysis; original values are shown in parentheses.

Weed control treatments	*Amaranthus spinosus *	*Celosia* *argentea *	*Cleome* *rutidosperma *	*Cyperus rotundus *	*Cynodon dactylon *	*Echinochloa colona *	Total weed density
Partial weedy	5.44 (29.2)	9.06 (85.4)	6.22 (38.5)	8.86 (79.0)	3.83 (14.6)	8.77 (79.2)	18.00 (325.8)
Oxadiargyl fb ethoxysulfuron	0.71 (0.0)	0.71 (0.0)	0.71 (0.0)	5.32 (28.1)	2.55 (6.3)	2.56 (8.3)	6.50 (42.7)
Pendimethalin fb ethoxysulfuron	0.71 (0.0)	4.26 (17.7)	0.71 (0.0)	6.07 (36.5)	2.15 (5.2)	1.97 (4.2)	7.97 (63.5)
Acetachlor + bensulfuranmethyl fb ethoxysulfuron	0.71 (0.0)	5.63 (31.3)	4.56 (20.8)	6.90 (47.9)	1.74 (3.1)	1.97 (4.2)	10.36 (107.3)
Pyrazosulfuron fb ethoxysulfuron	0.71 (0.0)	5.09 (26.0)	0.71 (0.0)	6.09 (37.5)	1.93 (6.3)	5.62 (31.3)	10.07 (101.0)
Oxadiargyl fb 1 HW at 35 DAS	0.71 (0.0)	0.71 (0.0)	0.71 (0.0)	7.90 (63.5)	1.93 (6.3)	0.71 (0.0)	8.30 (69.8)
Pendimethalin fb 1 HW at 35 DAS	0.71 (0.0)	5.24 (27.1)	0.71 (0.0)	9.40 (89.2)	1.52 (3.1)	0.71 (0.0)	10.93 (119.4)
Acetachlor + bensulfuraonmethyl fb 1 HW at 35 DAS	3.72 (13.5)	5.82 (33.3)	3.53 (12.5)	7.07 (50.0)	1.51 (2.1)	3.71 (13.5)	11.20 (125.0)
Pyrazosulfuron fb 1 HW at 35 DAS	3.67 (13.5)	6.14 (37.5)	0.71 (0.0)	7.54 (57.3)	3.96 (15.6)	7.47 (58.3)	13.42 (182.3)
LSD_0.05_	0.70	1.65	0.90	2.03	NS	2.14	2.08
*P* value	<0.001	<0.001	<0.001	0.009	0.20	<0.001	<0.001

DAS: days after sowing; fb: followed by; HW: hand weeding; LSD: least significant difference at 5% level of significance; *P*: probability.

**Table 3:** Effect of weed control treatments on weed density (number m^−2^) at 35 DAS in the aman season. Weed density data were subjected to square-root $\left(\sqrt{\left[x+0.5\right]}\right)$ transformation before analysis; original values are shown in parentheses.

Weed control treatments	*Ageratum conyzoides *	*Cyperus rotundus *	*Celosia* *argentea *	*Digitaria ciliaris *	*Echinochloa colona *	*Phyllanthus* *niruri *	Total weed density
Partial weedy	5.85 (34.4)	7.36 (54.2)	4.19 (17.7)	10.02 (102)	8.03 (64.2)	4.56 (20.8)	17.12 (293.3)
Oxadiargyl fb ethoxysulfuron	0.71 (0.0)	3.7 (13.6)	1.11 (1.1)	6.63 (43.8)	4.69 (21.8)	0.71 (0.0)	9.20 (84.3)
Pendimethalin fb ethoxysulfuron	0.71 (0.0)	3.60 (16.7)	4.12 (16.7)	5.99 (38.5)	4.86 (23.1)	3.88 (14.6)	10.74 (114.8)
Acetachlor + bensulfuranmethyl fb ethoxysulfuron	0.71 (0.0)	3.01 (11.5)	3.87 (14.6)	8.60 (74)	6.71 (45.8)	0.71 (0.0)	12.09 (145.8)
Pyrazosulfuron fb ethoxysulfuron	0.71 (0.0)	2.15 (8.3)	1.11 (1.1)	9.00 (85.4)	7.60 (57.3)	0.71 (0.0)	12.31 (152.1)
Oxadiargyl fb 1 HW at 35 DAS	2.35 (10.4)	7.38 (54.2)	1.74 (3.1)	7.27 (56.3)	5.81 (33.3)	0.71 (0.0)	12.53 (157.3)
Pendimethalin fb 1 HW at 35 DAS	4.97 (25)	8.14 (66.7)	4.72 (21.9)	5.33 (28.2)	4.92 (24.0)	3.56 (12.5)	13.34 (178.1)
Acetachlor + bensulfuraonmethyl fb 1 HW at 35 DAS	1.81 (5.2)	5.72 (32.3)	5.44 (29.2)	8.34 (69.3)	8.26 (67.7)	0.71 (0.0)	14.10 (198.4)
Pyrazosulfuron fb 1 HW at 35 DAS	2.91 (10.4)	6.40 (41.7)	5.21 (27.1)	9.49 (90.4)	7.69 (61.5)	1.34 (2.1)	15.13 (233.1)
LSD_0.05_	2.65	2.81	1.26	3.10	1.65	0.85	2.09
*P* value	0.005	0.002	<0.001	0.05	0.001	<0.001	<0.001

DAS: days after sowing; fb: followed by; HW: hand weeding; LSD: least significant difference at 5% level of significance; *P*: probability.

**Table 4:** Effect of weed control treatments on weed biomass (g m^−2^) at 35 days after sowing in the boro season. Weed biomass data were subjected to square-root $\left(\sqrt{\left[x+0.5\right]}\right)$ transformation before analysis; original values are shown in parentheses.

Weed control treatments	*Amaranthus spinosus *	*Celosia argentea *	*Cleome* *rutidosperma *	*Cyperus rotundus *	*Cynodon dactylon *	*Echinochloa colona *	Total weed biomass
Partial weedy	1.82 (2.8)	2.19 (4.6)	1.35 (1.4)	3.24 (10.1)	1.00 (0.5)	2.25 (4.7)	4.92 (24.1)
Oxadiargyl fb ethoxysulfuron	0.71 (0.0)	0.71 (0.0)	0.71 (0.0)	2.19 (4.3)	0.86 (0.2)	0.89 (0.3)	2.31 (4.9)
Pendimethalin fb ethoxysulfuron	0.71 (0.0)	1.19 (0.9)	0.71 (0.0)	2.36 (5.1)	0.92 (0.4)	0.79 (0.1)	2.65 (6.6)
Acetachlor + bensulfuranmethyl fb ethoxysulfuron	0.71 (0.0)	1.24 (1.0)	1.39 (1.5)	2.07 (3.8)	1.01 (0.6)	0.80 (0.1)	2.74 (7.0)
Pyrazosulfuron fb ethoxysulfuron	0.71 (0.0)	1.23 (1.0)	0.71 (0.0)	1.85 (3.0)	1.09 (1.0)	1.15 (0.9)	2.51 (5.9)
Oxadiargyl fb 1 HW at 35 DAS	0.71 (0.0)	0.71 (0.0)	0.71 (0.0)	3.01 (8.6)	1.03 (0.8)	0.71 (0.0)	3.13 (9.4)
Pendimethalin fb 1 HW at 35 DAS	0.71 (0.0)	1.07 (0.6)	0.71 (0.0)	3.50 (11.9)	0.73 (0.0)	0.71 (0.0)	3.60 (12.6)
Acetachlor + bensulfuraonmethyl fb 1 HW at 35 DAS	1.22 (1.0)	1.40 (1.5)	0.84 (0.2)	2.42 (5.5)	0.76 (0.1)	0.96 (0.4)	3.02 (8.7)
Pyrazosulfuron fb 1 HW at 35 DAS	1.27 (1.1)	1.40 (1.5)	0.71 (0.0)	2.09 (3.9)	0.98 (0.5)	1.61 (2.3)	3.10 (9.3)
LSD_0.05_	0.16	0.44	0.20	0.52	NS	0.40	0.63
*P* value	<0.001	<0.001	<0.001	<0.001	0.84	<0.001	<0.001

DAS: days after sowing; fb: followed by; HW: hand weeding; LSD: least significant difference at 5% level of significance; *P*: probability.

**Table 5:** Effect of weed control treatments on weed biomass (g m^−2^) at 35 days after sowing in the aman season. Weed biomass data were subjected to square-root $\left(\sqrt{\left[x+0.5\right]}\right)$ transformation before analysis; original values are shown in parentheses.

Weed control treatments	*Ageratum conyzoides *	*Cyperus rotundus *	*Celosia* *argentea *	*Digitaria ciliaris *	*Echinochloa colona *	*Phyllanthus* *niruri *	Total weed biomass
Partial weedy	2.60 (6.3)	3.77 (13.8)	3.12 (9.3)	6.29 (39.1)	6.23 (38.4)	2.42 (5.6)	10.63 (112.4)
Oxadiargyl fb ethoxysulfuron	1.00 (0.7)	2.03 (4.1)	1.42 (2.5)	3.53 (12.1 )	3.69 (13.2)	0.71 (0.0)	5.74 (32.5)
Pendimethalin fb ethoxysulfuron	1.14 (1.2)	2.12 (5.1)	3.04 (8.8)	3.74 (14.0)	3.42 (11.3)	2.01 (3.7)	6.67 (44.1)
Acetachlor + bensulfuranmethyl fb ethoxysulfuron	0.71 (0.0)	1.78 (3.3)	2.79 (7.3)	5.50 (30.6)	4.65 (21.3)	0.71 (0.0)	7.90 (62.5)
Pyrazosulfuron fb ethoxysulfuron	0.71 (0.0)	1.45 (2.7)	1.27 (1.4)	5.77 (34.3)	5.03 (24.9)	0.71 (0.0)	7.97 (63.3)
Oxadiargyl fb 1 HW at 35 DAS	1.29 (1.9)	4.03 (16)	1.27 (1.3)	3.87 (14.7)	3.92 (14.9)	0.71 (0.0)	7.01 (48.7)
Pendimethalin fb 1 HW at 35 DAS	2.57 (6.4)	4.61 (20.9)	3.56 (12.2)	3.62 (13.0)	3.54 (12.5)	1.40 (1.5)	8.17 (66.3)
Acetachlor + bensulfuraonmethyl fb 1 HW at 35 DAS	0.71 (0.7)	3.24 (10.1)	4.49 (19.7)	5.36 (28.7)	5.45 (29.6)	0.71 (0.0)	9.41 (83.4)
Pyrazosulfuron fb 1 HW at 35 DAS	1.42 (1.8)	3.70 (13.3)	3.86 (14.5)	6.00 (35.7)	5.17 (27.9)	0.83 (0.2)	9.66 (80.1)
LSD_0.05_	1.00	1.45	0.91	1.44	1.29	0.83	0.98
*P* value	0.003	0.002	<0.001	0.001	0.003	<0.001	<0.001

DAS: days after sowing; fb: followed by; HW: hand weeding; LSD: least significant difference at 5% level of significance; *P*: probability.

**Table 6:** Effect of weed control treatments on weed density (number m^−2^) at anthesis in the boro season. Weed density data were subjected to square-root $\left(\sqrt{\left[x+0.5\right]}\right)$ transformation before analysis; original values are shown in parentheses.

Weed control treatments	*Anagalis arvensis *	*Cyperus rotundus *	*Cynodon dactylon *	*Digitaria ciliaris *	*Dactyloctenium aegyptium *	*Echinochloa colona *	*Eleusine* *indica *	*Galinsoga* *ciliata *	*Phyllanthus* *niruri *	Total weed density
Partial weedy	12.67 (161)	8.66 (74.8)	5.03 (25)	5.76 (32.7)	4.36 (18.8)	7.39 (56.3)	3.04 (11.5)	8.68 (78.1)	4.38 (18.8)	21.80 (477.3)
Oxadiargyl fb ethoxysulfuron	0.71 (0.0)	8.56 (72.9)	7.73 (60.4)	4.26 (18.8)	0.71 (0.0)	2.35 (10.4)	0.71 (0.0)	2.40 (5.5)	0.71 (0.0)	12.98 (167.9)
Pendimethalin fb ethoxysulfuron	0.71 (0.0)	9.37 (89.6)	7.03 (49)	4.66 (21.5)	0.71 (0.0)	3.93 (15.6)	0.71 (0.0)	2.22 (4.7)	2.78 (9.4)	13.78 (189.7)
Acetachlor + bensulfuranmethyl fb ethoxysulfuron	0.71 (0.0)	10.14 (105)	7.79 (60.4)	4.86 (24.0)	0.71 (0.0)	5.09 (26.0)	0.71 (0.0)	0.71 (0.0)	0.71 (0.0)	14.58 (215.6)
Pyrazosulfuron fb ethoxysulfuron	0.71 (0.0)	8.46 (72.9)	7.07 (54.2)	6.42 (41.7)	2.33 (6.3)	6.52 (43.8)	4.48 (20.8)	0.71 (0.0)	2.91 (8)	15.75 (247.6)
Oxadiargyl fb 1 HW at 35 DAS	5.95 (35.4)	8.34 (70.4)	7.09 (50)	4.63 (30.2)	0.71 (0.0)	2.49 (5.8)	0.71 (0.0)	5.99 (37.5)	0.71 (0.0)	15.14 (229.3)
Pendimethalin fb 1 HW at 35 DAS	4.24 (18.1)	11.54 (133)	5.71 (32.3)	4.24 (17.7)	0.71 (0.0)	1.79 (3.3)	0.71 (0.0)	5.91 (35.4)	2.88 (8.3)	15.46 (238.4)
Acetachlor + bensulfuraonmethyl fb 1 HW at 35 DAS	5.63 (31.3)	10.35 (109)	6.19 (38.5)	4.60 (29.2)	0.71 (0.0)	3.99 (16.7)	0.71 (0.0)	4.92 (24.0)	0.71 (0.0)	15.77 (248.9)
Pyrazosulfuron fb 1 HW at 35 DAS	0.71 (0.0)	6.36 (43.5)	6.43 (45.8)	6.50 (42.7)	3.74 (13.5)	7.47 (67.8)	3.12 (9.4)	5.10 (25.7)	3.12 (9.4)	15.93 (257.8)
LSD_0.05_	1.07	2.49	NS	NS	0.91	3.67	1.36	1.71	1.24	2.84
*P* value	<0.001	0.03	0.42	0.63	<0.001	0.007	<0.001	<0.001	<0.001	<0.001

DAS: days after sowing; fb: followed by; HW: hand weeding; LSD: least significant difference at 5% level of significance; *P*: probability.

**Table 7:** Effect of weed control treatments on weed density (number m^−2^) at anthesis in the aman season. Weed density data were subjected to square-root $\left(\sqrt{\left[x+0.5\right]}\right)$ transformation before analysis; original values are shown in parentheses.

Weed control treatments	*Ageratum conyzoides *	*Cyperus rotundus *	*Digitaria ciliaris *	*Dactyloctenium aegyptium *	*Echinochloa colona *	*Eleusine indica *	*Galinsoga ciliata *	*Phyllanthus* *niruri *	Total weed density
Partial weedy	4.67 (21.7)	4.68 (24.0)	7.72 (59.6)	2.55 (6.3)	10.13 (102)	2.89 (8.1)	10.62 (117)	6.25 (39.8)	19.45 (378.3)
Oxadiargyl fb ethoxysulfuron	2.11 (4.0)	0.71 (0.0)	5.14 (26.0)	0.71 (0.0)	7.61 (57.5)	0.71 (0.0)	3.43 (11.7)	3.96 (15.6)	10.74 (114.8)
Pendimethalin fb ethoxysulfuron	1.38 (2.3)	0.71 (0.0)	6.01 (35.8)	0.71 (0.0)	6.54 (42.5)	2.94 (8.2)	5.75 (32.7)	5.14 (26.0)	12.16 (147.6)
Acetachlor + bensulfuranmethyl fb ethoxysulfuron	0.71 (0.0)	0.71 (0.0)	5.97 (35.4)	2.95 (10.4)	9.05 (81.7)	0.71 (0.0)	4.48 (21.0)	5.62 (31.3)	13.42 (179.8)
Pyrazosulfuron fb ethoxysulfuron	0.71 (0.0)	0.71 (0.0)	7.69 (64.6)	2.52 (6.1)	10.17 (104)	3.00 (8.7)	4.35 (20.3)	5.15 (27.3)	15.19 (230.7)
Oxadiargyl fb 1 HW at 35 DAS	2.81 (7.5)	2.79 (7.5)	4.68 (21.9)	0.71 (0.0)	4.60 (20.8)	0.71 (0.0)	10.05 (101)	4.38 (18.8)	13.32 (177.1)
Pendimethalin fb 1 HW at 35 DAS	2.49 (7.3)	3.41 (11.5)	5.52 (30.0)	0.71 (0.0)	6.36 (40.0)	0.71 (0.0)	9.40 (88.1)	4.75 (24.8)	14.22 (201.7)
Acetachlor + bensulfuraonmethyl fb 1 HW at 35 DAS	2.60 (6.3)	3.63 (13.4)	4.65 (21.9)	2.00 (4.3)	9.45 (90.6)	1.58 (2.4)	9.91 (98.3)	5.90 (35.4)	16.50 (272.6)
Pyrazosulfuron fb 1 HW at 35 DAS	2.55 (6.3)	3.97 (15.4)	3.25 (10.4)	1.92 (4.2)	9.88 (99.0)	2.32 (5.2)	9.10 (83.3)	6.43 (41.5)	16.30 (265.3)
LSD_0.05_	1.34	1.47	2.16	1.47	1.70	0.77	2.42	NS	1.07
*P* value	<0.001	<0.001	0.029	0.01	<0.001	0.001	<0.001	0.12	<0.001

DAS: days after sowing; fb: followed by; HW: hand weeding; LSD: least significant difference at 5% level of significance; *P*: probability.

**Table 8:** Effect of weed control treatments on weed biomass (g m^−2^) at anthesis in the boro season. Weed biomass data were subjected to square-root $\left(\sqrt{\left[x+0.5\right]}\right)$ transformation before analysis; original values are shown in parentheses.

Weed control treatments	*Anagalis arvensis *	*Cyperus rotundus *	*Cynodon dactylon *	*Digitaria ciliaris *	*Dactyloctenium aegyptium *	*Echinochloa colona *	*Eleusine indica *	*Phyllanthus niruri *	*Galinsoga ciliata *	Total weed biomass
Partial weedy	7.10 (52)	4.67 (21.7)	3.65 (12.9)	3.64 (13)	4.12 (19)	5.26 (27)	3.16 (13)	4.03 (16)	7.44 (56.0)	15.19 (230.2)
Oxadiargyl fb ethoxysulfuron	0.71 (0.0)	5.89 (35.3)	6.59 (42.9)	3.35 (11.1)	0.71 (0.0)	1.92 (6.1)	0.71 (0.0)	0.71 (0.0)	2.85 (7.9)	10.19 (103.3)
Pendimethalin fb ethoxysulfuron	0.71 (0.0)	6.64 (44.3)	5.54 (30.3)	3.82 (14.5)	0.71 (0.0)	2.75 (7.8)	0.71 (0.0)	2.60 (8.1)	2.90 (8.1)	10.66 (113.1)
Acetachlor + bensulfuranmethyl fb ethoxysulfuron	0.71 (0.0)	6.56 (43.7)	6.16 (37.5)	4.50 (20.3)	0.71 (0.0)	4.61 (21)	0.71 (0.0)	0.71 (0.0)	0.71 (0.0)	11.06 (122.8)
Pyrazosulfuron fb ethoxysulfuron	0.71 (0.0)	5.38 (27.7)	6.15 (41.7)	4.75 (22.1)	2.10 (4.9)	6.09 (39)	4.09 (17)	2.64 (6.5)	0.71 (0.0)	12.62 (158.9)
Oxadiargyl fb 1 HW at 35 DAS	3.23 (10.1)	6.07 (36.4)	5.44 (29.1)	2.51 (7.4)	0.71 (0.0)	2.59 (6.2)	0.71 (0.0)	0.71 (0.0)	6.70 (44.4)	11.58 (133.7)
Pendimethalin fb 1 HW at 35 DAS	3.30 (11)	6.75 (45.3)	4.55 (20.6)	3.41 (11.3)	0.71 (0.0)	2.26 (4.8)	0.71 (0.0)	2.34 (5.6)	6.64 (44.1)	11.96 (142.7)
Acetachlor + bensulfuraonmethyl fb 1 HW at 35 DAS	3.92 (14.9)	6.18 (38.4)	4.86 (24.4)	3.73 (18.3)	0.71 (0.0)	2.99 (8.9)	0.71 (0.0)	0.71 (0.0)	5.30 (28.3)	11.56 (133.2)
Pyrazosulfuron fb 1 HW at 35 DAS	0.71 (0.0)	5.03 (24.8)	5.31 (28.5)	5.61 (31.8)	3.70 (13.2)	5.98 (38.5)	3.62 (13.1)	2.57 (6.2)	6.28 (39)	13.97 (195.1)
LSD_0.05_	1.11	1.90	NS	NS	1.23	2.45	1.34	1.30	1.55	1.24
*P* value	<0.001	0.006	0.15	0.11	<0.001	0.001	<0.001	<0.001	<0.001	<0.001

DAS: days after sowing; fb: followed by; HW: hand weeding; LSD: least significant difference at 5% level of significance; *P*: probability.

**Table 9:** Effect of weed control treatments on weed biomass (g m^−2^) at anthesis in the aman season. Weed biomass data were subjected to square-root $\left(\sqrt{\left[x+0.5\right]}\right)$ transformation before analysis; original values are shown in parentheses.

Weed control treatments	*Ageratum conyzoides *	*Cyperus rotundus *	*Digitaria ciliaris *	*Dactyloctenium aegyptium *	*Echinochloa colona *	*Eleusine indica *	*Galinsoga ciliata *	*Phyllanthus* *niruri *	Total weed biomass
Partial weedy	2.99 (8.6)	2.67 (7.3)	7.11 (50.6)	1.78 (2.8)	9.09 (82.6)	2.01 (3.6)	7.12 (51.1)	5.59 (31.3)	15.43 (237.8)
Oxadiargyl fb ethoxysulfuron	2.24 (4.7)	0.71 (0.0)	5.37 (28.5)	0.71 (0.0)	8.00 (63.5)	0.71 (0.0)	2.59 (6.4)	3.28 (10.3)	10.67 (113.5)
Pendimethalin fb ethoxysulfuron	1.85 (3.6)	0.71 (0.0)	5.34 (28.10)	0.71 (0.0)	6.76 (45.6)	2.06 (3.8)	4.29 (18.2)	4.94 (24.0)	11.09 (123.1)
Acetachlor + bensulfuranmethyl fb ethoxysulfuron	0.71 (0.0)	0.71 (0.0)	4.54 (21.0)	2.28 (5.5)	9.46 (89.6)	0.71 (0.0)	3.48 (12.9)	4.01 (16.2)	12.04 (145.2)
Pyrazosulfuron fb ethoxysulfuron	0.71 (0.0)	0.71 (0.0)	6.88 (47.9)	1.98 (3.5)	10.08 (101.5)	2.12 (4.0)	4.18 (19.3)	3.88 (14.7)	13.83 (190.8)
Oxadiargyl fb 1 HW at 35 DAS	2.21 (4.4)	1.65 (2.3)	4.15 (17.1)	0.71 (0.0)	3.92 (15.3)	0.71 (0.0)	6.39 (40.5)	3.23 (10.2)	9.45 (89.7)
Pendimethalin fb 1 HW at 35 DAS	1.70 (2.9)	1.97 (3.6)	4.83 (23.2)	0.71 (0.0)	5.40 (28.7)	0.71 (0.0)	5.84 (33.7)	4.01 (17.1)	10.46 (109.2)
Acetachlor + bensulfuraonmethyl fb 1 HW at 35 DAS	1.77 (2.8)	1.94 (3.40)	3.50 (11.8)	1.49 (2.0)	7.19 (51.5)	1.39 (1.7)	5.69 (32.6)	4.89 (24.2)	11.42 (130.1)
Pyrazosulfuron fb 1 HW at 35 DAS	1.98 (3.5)	1.95 (3.3)	3.48 (11.6)	1.52 (2.1)	8.25 (68.5)	1.62 (2.4)	5.16 (26.4)	5.11 (25.6)	11.99 (143.5)
LSD_0.05_	0.89	0.76	1.33	0.87	1.39	0.55	1.88	1.46	1.30
*P* value	0.001	<0.001	<0.001	0.004	<0.001	<0.001	0.002	0.03	<0.001

DAS: days after sowing; fb: followed by; HW: hand weeding; LSD: least significant difference at 5% level of significance; *P*: probability.

**Table 10:** Effect of weed control treatments on yield and yield components of rice in the boro season.

Weed control treatments	Panicles (no. m^−2^)	Spikelet panicle^−1^	Spikelet fertility (%)	1000-grain weight (g)	Grain yield (t ha^−1^)
Weed-free	347.00	82.35	93.52	19.02	4.76
Partial weedy	159.67	59.33	92.79	18.03	1.58
Oxadiargyl fb ethoxysulfuron	315.67	78.38	93.67	18.70	4.13
Pendimethalin fb ethoxysulfuron	301.00	80.25	92.49	18.54	4.08
Acetachlor + bensulfuranmethyl fb ethoxysulfuron	303.00	77.93	92.77	18.61	4.01
Pyrazosulfuron fb ethoxysulfuron	281.67	76.05	91.74	18.05	3.46
Oxadiargyl fb 1 HW at 35 DAS	278.33	76.42	91.67	18.36	3.48
Pendimethalin fb 1 HW at 35 DAS	264.00	76.12	91.90	18.23	3.38
Acetachlor + bensulfuraonmethyl fb 1 HW at 35 DAS	270.33	76.62	91.99	18.90	3.42
Pyrazosulfuron fb 1 HW at 35 DAS	248.33	75.47	90.00	18.62	3.22
LSD_0.05_	23.41	5.65	1.63	NS	0.28
*P* value	*P* < 0.001	*P* < 0.001	0.008	0.074	*P* < 0.001

DAS: days after sowing; fb: followed by; HW: hand weeding; LSD: least significant difference at 5% level of significance; *P*: probability.

**Table 11:** Effect of weed control treatments on yield and yield components of rice in the aman season.

Weed control treatments	Panicles (no. m^−2^)	Spikelet panicle^−1^	Spikelet fertility (%)	1000-grain weight (g)	Grain yield (t ha^−1^)
Weed-free	253.33	120.20	88.71	17.79	4.98
Partial weedy	172.33	90.28	88.93	17.97	2.59
Oxadiargyl fb ethoxysulfuron	230.00	102.30	91.27	17.55	3.53
Pendimethalin fb ethoxysulfuron	219.67	102.52	88.43	17.74	3.43
Acetachlor + bensulfuranmethyl fb ethoxysulfuron	186.67	100.57	85.44	17.19	3.07
Pyrazosulfuron fb ethoxysulfuron	180.33	95.77	89.53	16.72	3.00
Oxadiargyl fb 1 HW at 35 DAS	236.33	112.68	86.63	18.17	4.08
Pendimethalin fb 1 HW at 35 DAS	230.67	113.63	87.57	18.28	4.05
Acetachlor + bensulfuraonmethyl fb 1 HW at 35 DAS	217.33	110.70	89.99	17.07	3.95
Pyrazosulfuron fb 1 HW at 35 DAS	204.00	107.97	86.29	17.00	3.85
LSD_0.05_	19.30	10.71	2.55	NS	0.19
*P* value	<0.001	0.001	0.002	0.32	<0.001

DAS: days after sowing; fb: followed by; HW: hand weeding; LSD: least significant difference at 5% level of significance; *P*: probability.
